# Loop diuretics inhibit kynurenic acid production and kynurenine aminotransferases activity in rat kidneys

**DOI:** 10.1007/s43440-024-00648-8

**Published:** 2024-09-11

**Authors:** Izabela Zakrocka, Katarzyna M. Targowska-Duda, Tomasz Kocki, Waldemar Turski, Ewa M. Urbańska, Wojciech Załuska

**Affiliations:** 1 Department of Nephrology, Medical University of Lublin, Jaczewskiego 8, 20-954 Lublin, Poland; 2 Department of Biopharmacy, Medical University of Lublin, Chodźki 4a, 20-093 Lublin, Poland; 3 Department of Experimental and Clinical Pharmacology, Medical University of Lublin, Jaczewskiego 8b, 20-090 Lublin, Poland

**Keywords:** Kynurenic acid, Kynurenine, Tryptophan, Kynurenine pathway, Kidney, Loop diuretics

## Abstract

**Background:**

Loop diuretics became a cornerstone in the therapy of hypervolemia in patients with chronic kidney disease or heart failure. Apart from the influence on water and electrolyte balance, these drugs were shown to inhibit tissue fibrosis and renin-angiotensin-system activity. The kynurenine (KYN) pathway products are suggested to be uremic toxins. Kynurenic acid (KYNA) is synthesized by kynurenine aminotransferases (KATs) in the brain and periphery. The cardiovascular and renal effects of KYNA are well documented. However, high KYNA levels have been correlated with the rate of kidney damage and its complications. Our study aimed to assess the effect of loop diuretics, ethacrynic acid, furosemide, and torasemide on KYNA synthesis and KATs activity in rat kidneys in vitro.

**Methods:**

Quantitative analyses of KYNA were performed using fluorimetric HPLC detection. Additionally, molecular docking studies determined the possible interactions of investigated compounds with an active site of KAT I and KAT II.

**Results:**

All studied drugs inhibited KYNA production in rat kidneys in vitro at 0.5–1.0 mmol/l concentrations. Only ethacrynic acid at 1.0 mmol/l concentration significantly lowered KAT I and KAT II activity in kidney homogenates, whereas other drugs were ineffective. Molecular docking results indicated the common binding site for each of the studied loop diuretics and KYNA. They suggested possible residues involved in their binding to the active site of both KAT I and KAT II model.

**Conclusions:**

Our study reveals that loop diuretics may decrease KYNA synthesis in rat kidneys in vitro. The presented results warrant further research in the context of KYN pathway activity regulation by loop diuretics.

**Graphical abstract:**

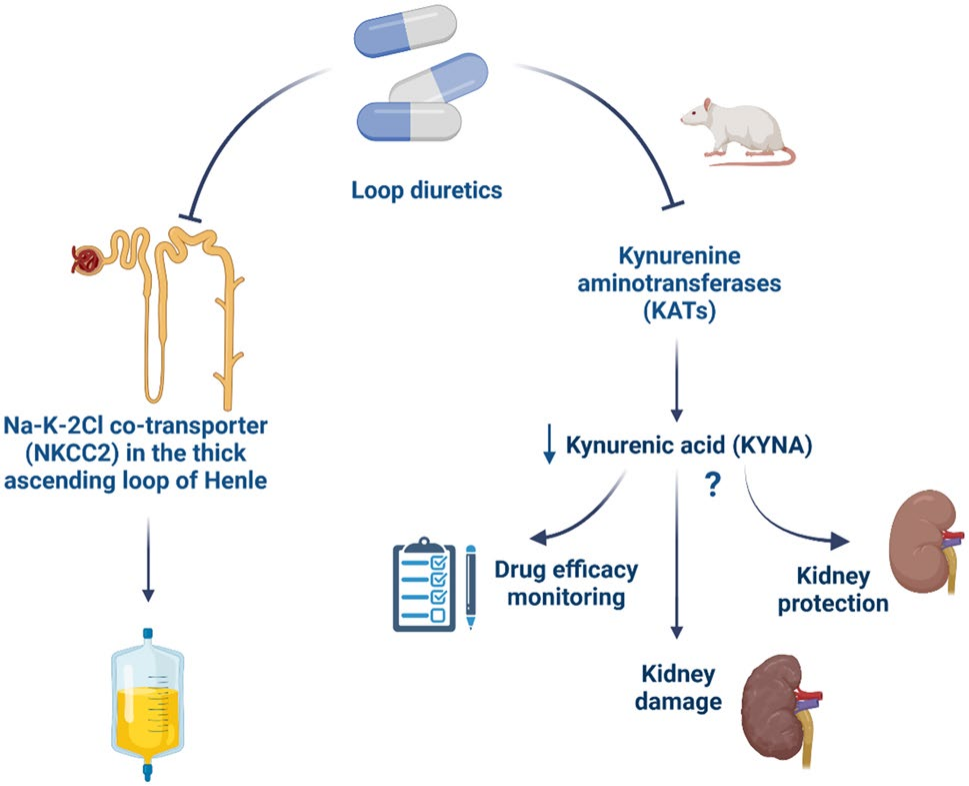

## Introduction

Water and electrolyte balance is vital for patients with heart failure, liver cirrhosis, or kidney diseases. Fluid overload is closely linked with frequency of hospitalizations, reduced quality of life, poor prognosis, and high costs of health care [1]. Therapy of congestion is complex, yet loop diuretics, due to their high efficacy and rather good bioavailability, remain the first-line treatment of overhydration [2]. Upon secretion to the proximal tubule, loop diuretics bind to sodium/potassium/chloride cotransporter 2 (NKCC2) at the luminal membrane of the thick ascending loop of Henle, which reduces sodium reabsorption and stimulates vigorous natriuresis [3]. However, a secondary increase of sodium chloride reabsorption in the distal convoluted tubule develops and contributes to nephron remodeling. Loop diuretics may also impair tubuloglomerular feedback thus preventing the decline of glomerular filtration. Competitive inhibition of organic anion transporter 4 (OAT4), multidrug resistance protein 4 (MRP4), and human sodium phosphate transporter 4 (hNPT4) by loop diuretics were shown in the proximal tubules [4–6]. OATs play a major role in loop diuretics secretion in the proximal tubule [7, 8], and take part in transporter-related drug interactions [9].

Loop diuretics may also decrease intramedullary prostaglandin synthesis and medullary perfusion [10]. Commonly used loop diuretics include furosemide, torasemide, and ethacrynic acid. Furosemide displays variable bioavailability and a short half-life, yet manifests a fast onset of action, whereas torasemide is more preferred in long-term treatment due to higher bioavailability, longer duration of action, and lower dependence on kidney function [11]. Importantly, contrary to other class representatives, torasemide was shown to inhibit the renin-angiotensin-aldosteron system (RAAS), with beneficial effects on tissue remodeling [12]. Ethacrynic acid, the first registered representative of loop diuretics, is used as an alternative for patients not tolerating sulfonamide-containing drugs [11].

Although data about loop diuretics' effect on kidney function, especially in the case of acute kidney injury (AKI) remain controversial, some studies suggest the nephroprotective potential of this class of drugs, mainly due to antiapoptotic effect and suppression of angiogenesis-related genes [13]. On the other hand, it has been postulated that loop diuretics may impair kidney function, possibly through the deterioration of oxygen utilization in the ischemia–reperfusion injury model [14] or inhibition of mitochondrial activity [15].

The kynurenine (KYN) pathway is a major route of tryptophan metabolism, resulting in the formation of numerous biologically active substances [16]. KYN metabolites are involved in various physiological processes, such as cellular survival, immune response, or cardiovascular and kidney function. Kynurenic acid (KYNA), produced in the brain and the periphery mostly through enzymatic conversion of KYN by kynurenine aminotransferases (KATs), KAT I and KAT II, is excreted in large quantities by kidneys [17]. KYNA targets glutamate receptors [18], blocks α7 nicotinic acetylcholine receptors [19], activates aryl hydrocarbon receptors (AhRs) [20], hydroxycarboxylic acid receptor 3 (HCAR3) [21], and is a ligand of G protein-coupled receptor 35 (GPR35) [22]. In the brain, KYNA exerts neuroprotective and anticonvulsant effects. Disturbed metabolism of KYNA has been therefore implicated in the pathogenesis of multiple neuropsychiatric conditions [23]. In the periphery, natriuretic [24] and chronotropic negative effects in spontaneously hypertensive rats were shown [25]. Through GPR35 KYNA may also inhibit mitochondrial damage and reactive oxygen species production, resulting in NLRP3 inflammasome blockade [26]. Decreased KYNA removal due to impaired kidney function results in its significant accumulation in body fluids. Lower KYNA clearance was associated with chronic kidney disease (CKD) complications, namely hyperparathyroidism, hypertriglyceridemia [27], and cardiovascular events [28], independently of kidney function or albuminuria level. Several groups of drugs, in particular hypoglycemic [29], hypolipidemic [30], anti-inflammatory [31], and antihypertensive agents [32, 33] have been presented as inhibitors of KYNA synthesis, with a possible direct effect on kidney function.

Since KYNA was shown to take part in regulating water and electrolyte balance, and its production can be decreased by hypotensive drugs, the goal of this study was to analyze the effect of loop diuretics: ethacrynic acid, furosemide and torasemide, on KYNA synthesis and KATs activity in rat kidney in vitro. Additionally, molecular docking was performed to evaluate the possibility of binding of tested compounds to the active site of KAT I and KAT II.

## Materials and methods

### Animals

The experiments were performed on tissue obtained from male adult Wistar rats kept in the Experimental Medicine Center, Medical University of Lublin, Poland. Animals weighing 180–220 g were housed under standard laboratory conditions (temperature 20 °C; 12-h light–dark cycles), with food and water available ad libitum. Animals were used after 7 days of adaptation. The experiments were conducted between 9.00 a.m. and 1.00 p.m. All experiments were performed according to the National Institute of Health Guidelines for the Care and Use of Laboratory Animals (8th edition), the European Community Council Directive for the Care and Use of Laboratory Animals of 22 September 2010 (2010/63/EU) and with ARRIVE guidelines. During planning of experiments all efforts were made to maintain animals welfare protection according to the 3Rs rule. Kidneys from 6 animals were used in this study to obtain comparable results. To avoid interference of anesthetics with KYNA synthesis and KATs activity no anesthesia was used, kidneys were harvested after animals rapid decapitation. According to current Polish and European legislation, the removal of organs or cells from vertebrates for scientific purposes is not considered an animal experiment if the animals have not been subject to surgical interventions or invasive treatments prior to sacrifice. Consequently, the euthanasia of rat intended for the removal of brain tissue and all of the further procedures do not necessitate the approval or permission of local or governmental authorities.

### Substances

l-Kynurenine (sulfate salt) (K3750), tested drugs: ethacrynic acid (SML1083), furosemide (F4381), and torasemide (T3202); reagents used to prepare Krebs Ringer buffer: sodium chloride (S7653), potassium chloride (P9333), magnesium sulfate heptahydrate (M7506), calcium chloride anhydrous (C1016), sodium phosphate monobasic dihydrate (71,505), sodium phosphate dibasic (S0876), glucose (G8270), distilled water; drugs solvent: dimethyl sulfoxide (DMSO) (D1435); reagents necessary for KATs analysis conduction: Trizma base (T1503), acetic acid (A6283), pyridoxal 5′-phosphate hydrate (P9255), 2-mercaptoethanol (M3148), sodium pyruvate (P2256), and d-glutamine (D9003) were purchased from Sigma-Aldrich. Compounds essential for high-performance liquid chromatography (HPLC) were obtained from J.T. Baker Chemicals and Sigma-Aldrich. All examined drugs were dissolved in the DMSO, with a final DMSO concentration not higher than 5% [34].

### KYNA synthesis in rat kidney homogenates in vitro

Both kidneys were collected immediately after the decapitation of each animal and transferred into the ice-cold bath (+ 4 °C). Subsequently, organs were weighed and homogenized in freshly oxygenated Krebs–Ringer buffer pH 7.4 (1:4; w/v). Afterward, 100 μL of kidney homogenate was incubated in oxygenated Krebs–Ringer buffer, for 2 h at 37 °C, in the presence of 2 µmol/l l-KYN and studied substances. Substances were tested in six different concentrations: 1 μmol/l, 10 μmol/l, 50 μmol/l, 100 μmol/l, 500 μmol/l, and 1 mmol/l, and six kidney samples were used for each concentration (N = 6). The procedure was terminated by a rapid transfer of samples into an ice bath and the addition of 100 μL of 1 N HCl into each tube. Samples were centrifuged (15,133×*g*, 15 min), and obtained supernatants were stored until further HPLC analysis. Presented experiments were performed twice, obtained results from both repetitions were similar.

### KATs activity in rat kidney homogenates in vitro

KAT isoenzymes activity evaluation in rat kidneys in vitro was adapted from Gramsbergen et al. [35]. In short, obtained rat kidneys were homogenized in dialysate buffer containing 5 mmol/l Tris–acetate buffer (at pH 8.0), 50 μmol/l pyridoxal 5′-phosphate, and 10 mmol/l 2-mercaptoethanol. Prepared kidney homogenate was centrifuged (15,133×*g*, 15 min), and collected supernatant was dialyzed against 4 l of the dialysate buffer for 12 h, at 8 °C, by using cellulose membrane dialysis tubing. Next, harvested semi-purified enzymatic preparation was incubated for 2 h, at 37 °C, with l-KYN (2 μmol/l), and analyzed drugs at 6 different concentrations (1 μmol/l to 1 mmol/l). To achieve maximal enzymatic activity, the pH of the reaction mixture was set at 9.5 and 7.0 for KAT I or KAT II activity analysis, respectively. The KAT I inhibitor, glutamine (2 mmol/l) was added to test tubes intended to assess the activity of KAT II. All reactions were stopped by transferring samples into an ice-cold bath. Samples were centrifuged and resulting supernatants were stored until further HPLC analysis. In vitro experiments were performed twice with technical triplicates. The results from both repetitions were similar.

### HPLC analyses

KYNA content in samples was quantified using HPLC analysis (Thermo Fisher Scientific HPLC system), as previously described by Shibata [36]. ESA catecholamine HR-80, 3 μm, C18 reverse-phase column was used. The mobile phase containing: 250 mmol/l zinc acetate, 25 mmol/l sodium acetate, 5% acetonitrile, and pH at 6.2 was run with a flow rate of 1.0 ml/min through the system. The fluorescence detector was set at the following parameters: excitation 344 nm, emission 398 nm.

### Molecular docking of ethacrynic acid, furosemide, torasemide, and kynurenine to KAT I and KAT II

The available crystal structure of the hKAT I in PMP form at 2.90 Å atomic resolution (PDB ID: 1W7N) [37] as well as hKAT II in complex with its substrate KYN and co-factor PMP at 1.95 Å atomic resolution (PDB ID: 2R2N) [38] was used to perform the molecular docking simulations. In the next step, ethacrynic acid, furosemide, and torasemide (Molfile) were imported from the PubChem Database and optimized using the semi-empirical method AM1 and then transferred for the subsequent step of ligand docking. Molegro Virtual Docker (v 6.0.0, Molegro ApS, Aarhus, Denmark) was used for docking simulations of flexible ligands into the rigid KAT I and KAT II structures. The docking parameters were used as previously described [33]. The lower energy conformations were selected from possible clusters of superposed poses for each studied ligand to both targets (KAT I and KAT II).

### Statistical analysis

The results of experiments on kidney homogenates and KATs are presented as mean ± standard deviation (SD), whereas results of KAT I and KAT II activity analyses are shown as median with interquartile range. Data analysis was carried out by the one-way analysis of variance (one-way ANOVA) followed by Tukey’s multiple comparison test (KYNA synthesis experiments) or Kruskal–Wallis test followed by *post-hoc* Dunn’s test (KAT I and KAT II activity experiments) in the GraphPad Prism 6. The *p-value* < 0.05 was established as statistically significant.

## Results

### Influence of loop diuretics on KYNA production in rat kidney in vitro

**Fig. 1:**
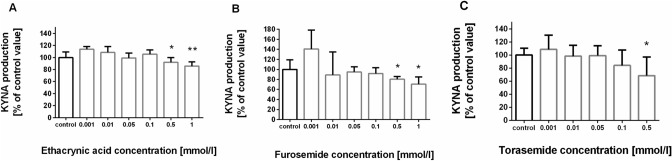
The influence of loop diuretics: ethacrynic acid (**A**), furosemide (**B**) and torasemide (**C**) on kynurenic acid (KYNA) production in rat kidney in vitro. Data are presented as a percentage of control KYNA production, mean ± SD, N = 6, *p < 0.05, **p < 0.01. ANOVA followed by Tukey’s multiple comparison test. Standard KYNA synthesis in tested rat kidney homogenates under 2 µmol/l l-kynurenine (l-KYN) was 1.72 ± 0.35 pmol/mg of fresh kidney tissue

Standard KYNA synthesis in tested rat kidney homogenates under 2 µmol/l l-KYN was 1.72 ± 0.35 pmol/mg of fresh kidney tissue. Ethacrynic acid at 0.5 mmol/l and 1 mmol/l lowered KYNA formation in rat kidney homogenates to 92% and 86% of control value, respectively (F_6,35_ = 8.911, *p* < 0.0001, ANOVA followed by Tukey’s multiple comparison test) (Fig. 1A). Similarly, furosemide inhibited KYNA synthesis in rat kidney in vitro at 0.5 mmol/l and 1 mmol/l concentration to 80% and 70% of control, respectively (F_6,35_ = 4.816, *p* = 0.0011, ANOVA followed by Tukey’s multiple comparison test) (Fig. 1B). Torasemide, decreased KYNA production in analyzed rat kidney homogenates at 0.5 mmol/l concentration to 68% of control (F_5,30_ = 3.041, *p* = 0.0245, ANOVA followed by Tukey’s multiple comparison test) (Fig. 1C).

### Influence of loop diuretics on KAT I activity in rat kidney in vitro

**Fig. 2:**
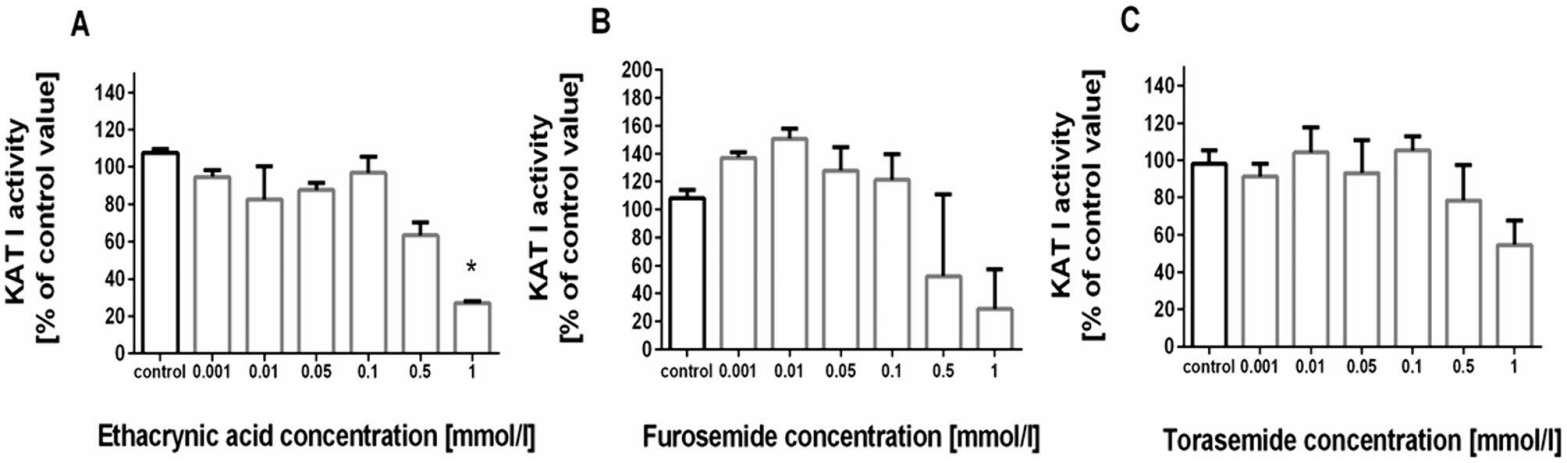
The influence of loop diuretics: ethacrynic acid (**A**), furosemide (**B**) and torasemide (**C**) on kynurenine aminotransferase I (KAT I) activity in rat kidney in vitro. Data are presented as a percentage of control kynurenic acid (KYNA) production by KAT I, median with interquartile ranges, N = 3, *p < 0.05. Kruskal–Wallis test followed by *post-hoc* Dunn’s test. KAT I activity in control samples in the presence of 2 µmol/l l-KYN was 82.58 ± 11.35 pmol/mg of protein

The mean production of KYNA in rat kidney semi-purified KAT I in the presence of 2 µmol/l l-KYN was 82.58 ± 11.35 pmol/mg of protein. Only ethacrynic acid at 1 mmol/l concentration significantly reduced KAT I activity in rat kidneys to 27% of control value (H = 12.73, N_1_ = 3, N_2_ = 3, N_3_ = 3, N_4_ = 3, N_5_ = 3, N_6_ = 3, N_7_ = 3, *p* = 0.0149, Kruskal–Wallis test followed by *post-hoc* Dunn’s test) (Fig. 2A). Other diuretics, furosemide (Fig. 2B) and torasemide (Fig. 2C) lowered KAT I activity in kidney homogenates, however results were statistically not significant.

### Influence of loop diuretics on KAT II activity in rat kidney in vitro

**Fig. 3:**
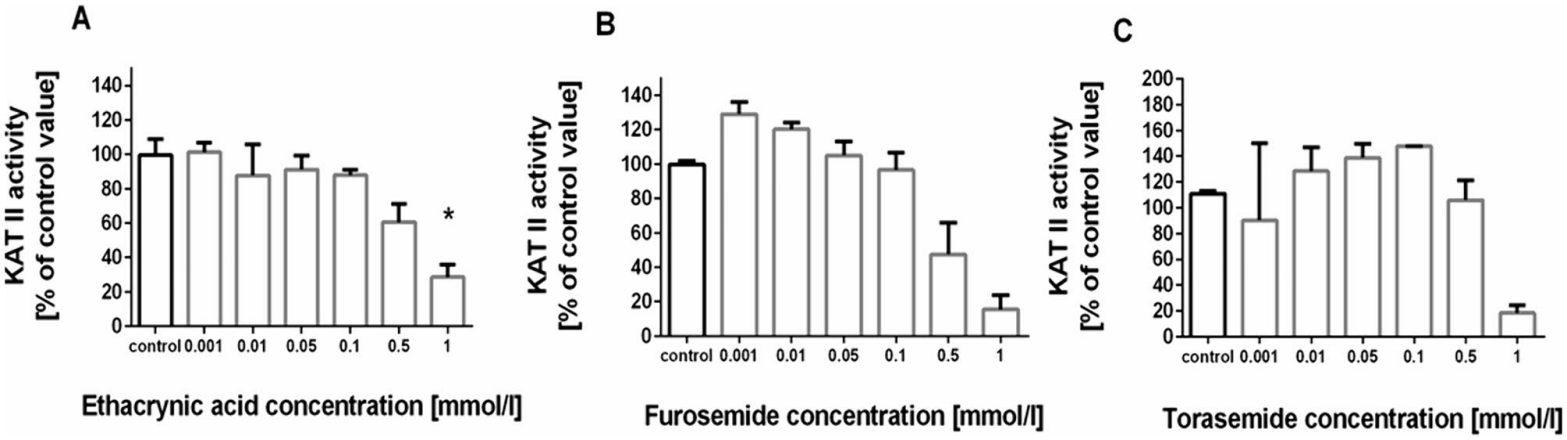
The influence of loop diuretics: ethacrynic acid (**A**), furosemide (**B**) and torasemide (**C**) on kynurenine aminotransferase II (KAT II) activity in rat kidney in vitro. Data are presented as a percentage of control kynurenic acid (KYNA) production by KAT II, median with interquartile ranges, N = 3, *p < 0.05. Kruskal–Wallis test followed by *post-hoc* Dunn’s test. KAT II activity in control rat kidney samples in the presence of 2 µmol/l l-KYN was 192 ± 73.58 pmol/mg of protein

KAT II activity in tested rat kidney in the presence of 2 µmol/l l-KYN was 192 ± 73.58 pmol/mg of protein. Ethacrynic acid lowered the activity of KAT II in rat kidney in vitro at 1 mmol/l concentration to 28% of control value (H = 12.83, N_1_ = 3, N_2_ = 3, N_3_ = 3, N_4_ = 3, N_5_ = 3, N_6_ = 3, N_7_ = 3, *p* = 0.0120, Kruskal–Wallis test followed by *post-hoc* Dunn’s test) (Fig. 3A). Furosemide (Fig. 3B) and torasemide (Fig. 3C) did not significantly decrease KAT II activity in kidney homogenates at all tested concentrations.

### Molecular docking of loop diuretics to KAT I and KAT II

**Fig. 4:**
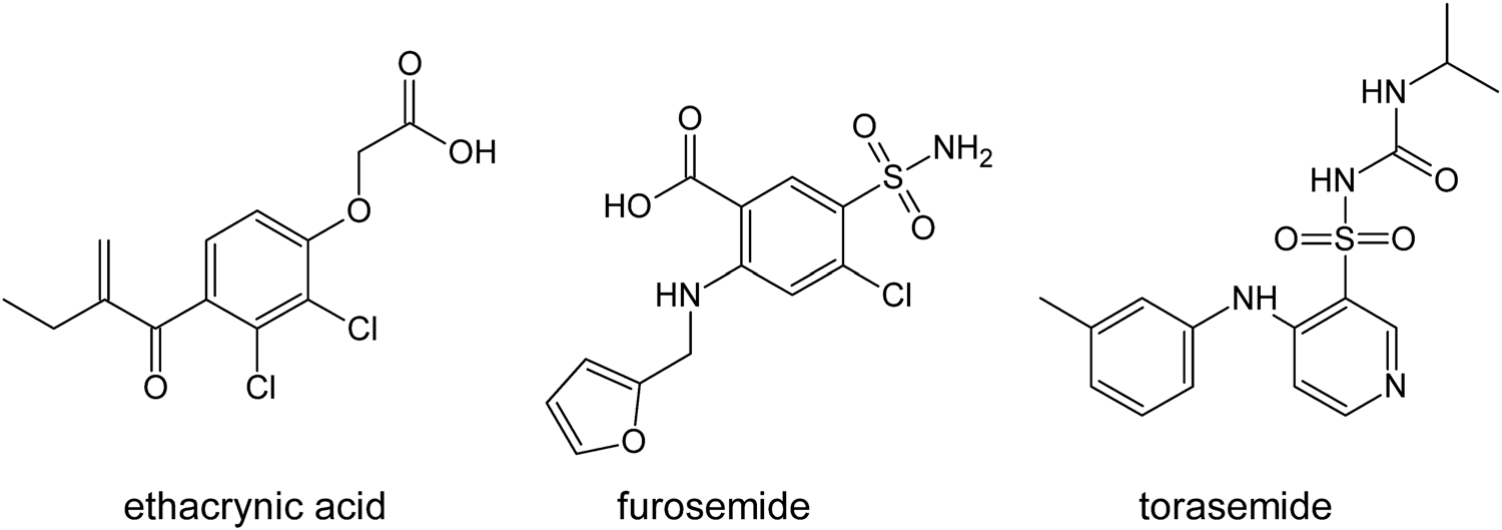
Molecular structures of ethacrynic acid, furosemide, and torasemide

**Fig. 5:**
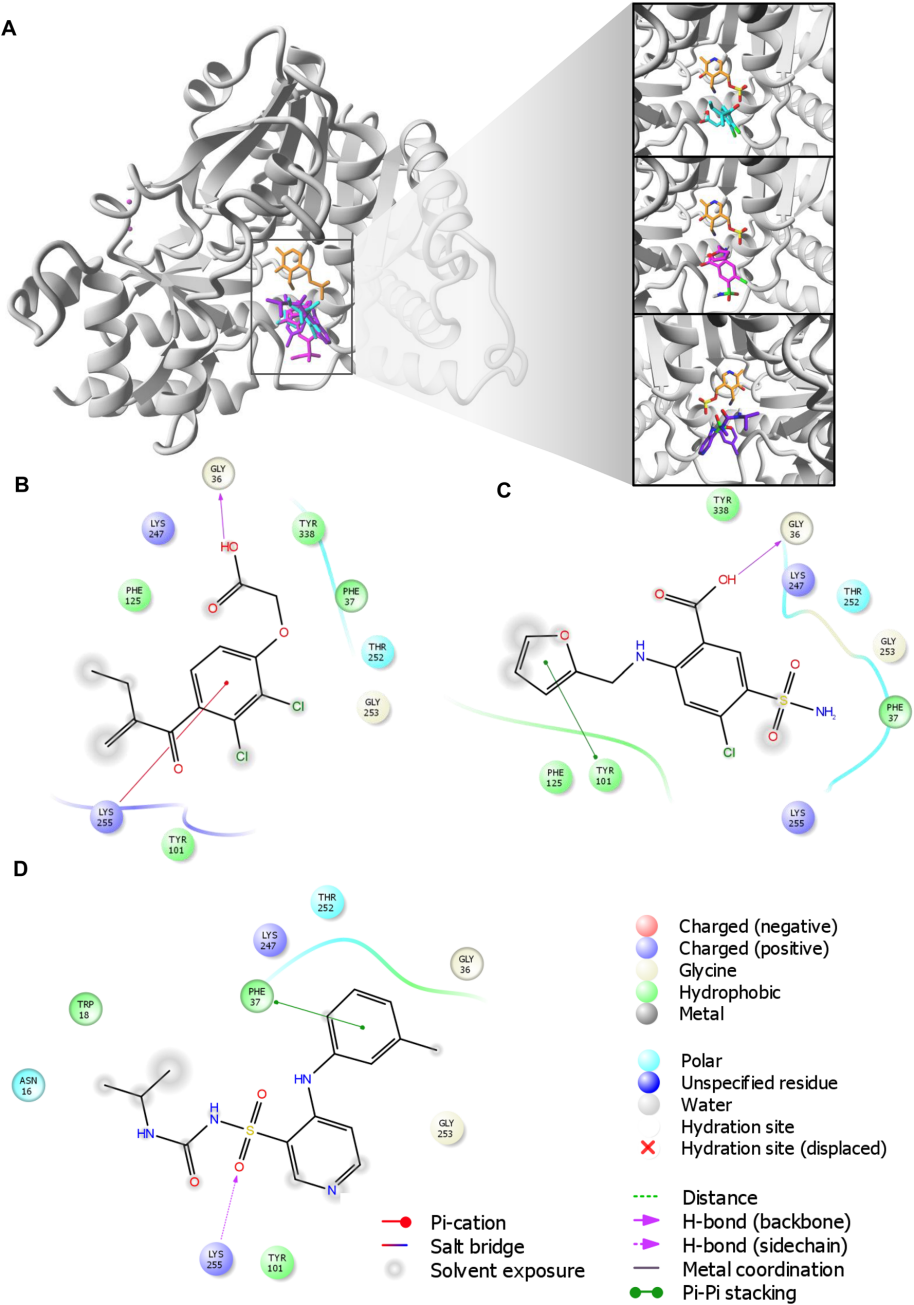
Molecular docking of loop diuretics to the crystal structure of kynurenine aminotransferase I (KAT I) (PDB ID: 1W7N). **A** All ligands binding sites, including ethacrynic acid (cyan) furosemide (magenta), and torasemide (purple) together with co-factor (orange) at enzyme active site. **B** 2D map for the residues involved in ethacrynic acid binding at energetically lowest orientation 1. **C** 2D map for the residues involved in furosemide binding at energetically lowest orientation 1. (D) 2D map for the residues involved in torasemide binding at energetically lowest orientation 1. Ligands are rendered in stick mode; KAT I (chain A from chain B) structure is shown in grey. Oxygen atoms are colored red, nitrogens blue, phosphorus yellow, hydrogen white, and chlorine green. All hydrogen atoms are hidden

The molecular docking results suggested that all examined loop diuretics (structures depicted in Fig. 4) bind to the active site of both KAT I and KAT II. More specifically, docking simulations of each ligand to the active site of KAT I suggested that KYN, ethacrynic acid, furosemide, and torasemide interact with common residues in the KAT I active site, including Gly36, Phe37, Tyr101, Phe125, Gly253, and Lys255 (Table 1 and Fig. 5). In addition, hydrogen bond was suggested between hydroxyl group of either ethacrynic acid or furosemide and side chain of Gly36. In the case of torasemide, the hydrogen bond was formed with Lys255. π–π interactions were suggested either between the furyl moiety of furosemide and Ty101 or between the benzyl moiety of torasemide and Phe37 (Fig. 5C, D, respectively). For ethacrynic acid, cation-π interactions were formed between phenyl moiety and Lys255 (Fig. 5B).

**Table 1:**
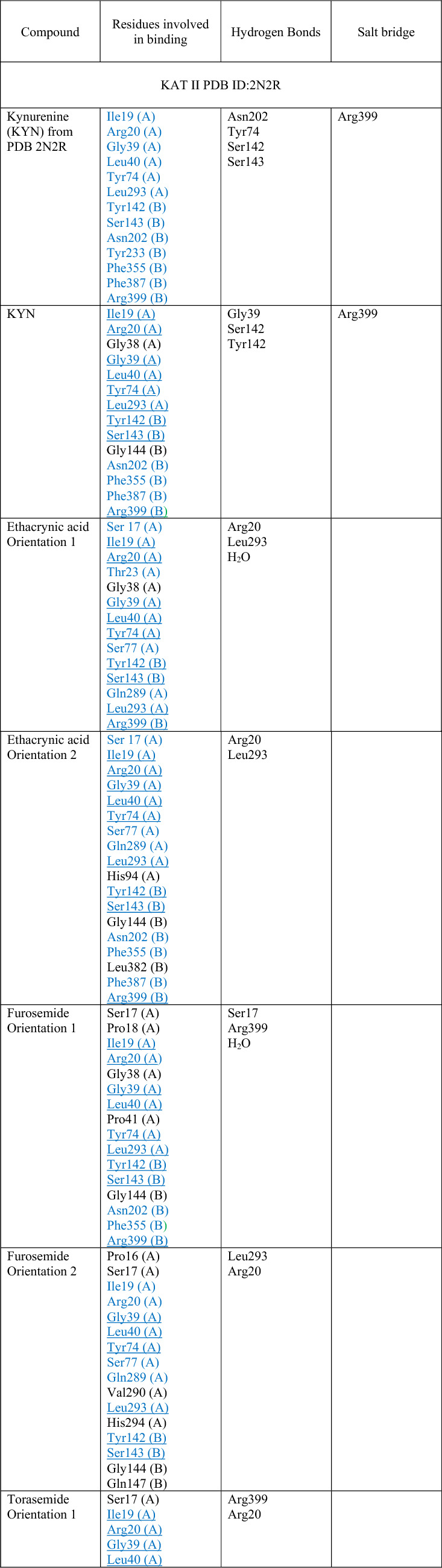

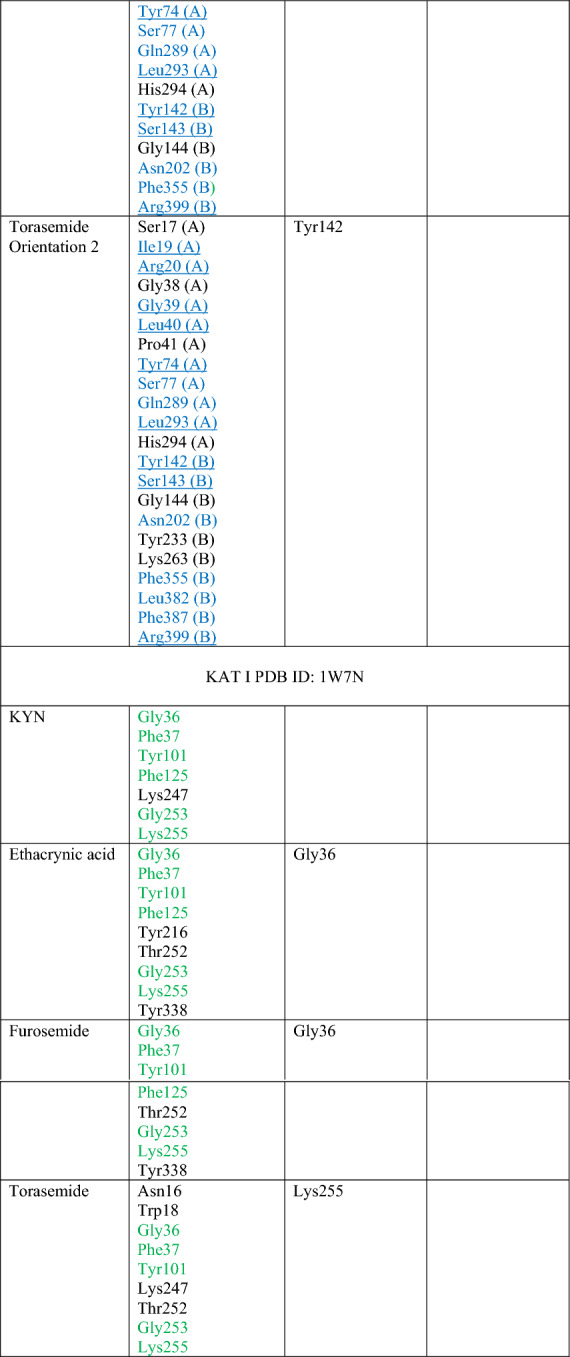
Molecular interactions of loop diuretics with kynurenine aminotransferase I (KAT I) and kynurenine aminotransferase II (KAT II) active site

Residues in blue are from the crystal structure of KAT II (PDN ID: 2R2N)

Underlined residues are common for all orientations presented for each studied ligand that interacts with KAT II

**Fig. 6:**
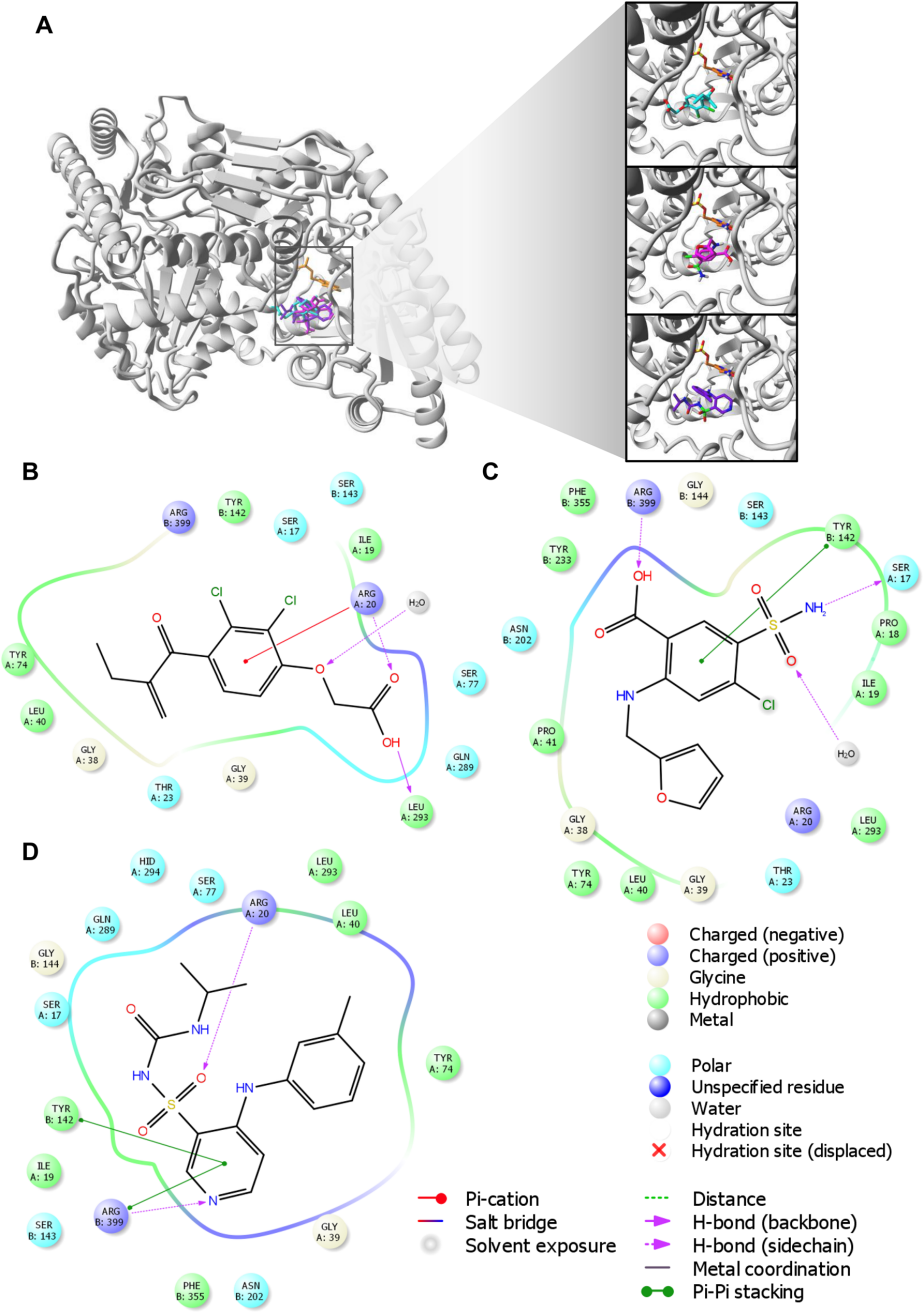
Molecular docking of loop diuretics to the crystal structure of kynurenine aminotransferase II (KAT II) (PDB ID: 2R2N). **A** All ligands binding sites, including ethacrynic acid (cyan) furosemide (magenta), and torasemide (purple) together with co-factor (orange) overlap the KYN binding pocket. **B** 2D map for the residues involved in ethacrynic acid binding at energetically lowest orientation 1. **C** 2D map for the residues involved in furosemide binding at energetically lowest orientation 1. **D** 2D map for the residues involved in torasemide binding at energetically lowest orientation 1. Ligands are rendered in stick mode; KAT II (chain A from chain B) structure is shown in grey. Oxygen atoms are colored red, nitrogens blue, phosphorus yellow, hydrogen white, and chlorine green. All hydrogen atoms are hidden

In the case of KAT II, a comparable position of KYN within the KAT II active site was found, consistent with its orientation in the three-dimensional crystal structure determined by Han et al. of KAT II [38] with KYN (PDB ID: 2R2N). In this regard, the residues indicated for KYN were also found as important for studied loop diuretics (i.e., ethacrynic acid, furosemide, and torasemide) interactions with KAT II, including Ile19 (A), Arg20 (A), Gly39 (A), Leu40 (A), Tyr74 (A), Leu293 (A) from one subunit, and Tyr142 (B), Ser143 (B), Asn202 (B), Tyr233 (B), Phe355 (B), Phe387 (B), and Arg399 (B) from the opposite subunit (see Table 1 for details). However, docking results suggested few orientations of each ligand in the KAT II active site, the main residues involved in binging were found similar for each orientation as included in Table 1 and shown for ethacrynic acid, furosemide, and torasemide in Fig. 6B–D, respectively. In the energetically lowest orientation of ethacrynic acid, three hydrogen bonds were suggested with its carboxyl group and Arg20, water molecule, and Leu293 (Fig. 6B). For the lowest conformations of furosemide and torasemide, two hydrogen bonds with Arg399 were suggested (Fig. 6C, D, respectively). Moreover, hydrogen bonds were formed between the sulfonamide group of furosemide and Ser17 or water molecule. In addition, π–π interactions were suggested between furyl moiety and Tyr142. Two possible hydrogen bonds were found between the sulfonamide group of torasemide and Arg20 or pyridine moiety of the ligand and Arg399 (Fig. 6D). π–π interactions were suggested between the pyridine moiety of torasemide and Arg399.

## Discussion

The presented study reveals that loop diuretics, ethacrynic acid, furosemide, and torasemide inhibit KYNA production in rat kidneys in vitro. Moreover, ethacrynic acid significantly inhibited the activity of renal KAT I and KAT II. The obtained data indicate that loop diuretics may inhibit the production of KYNA, and this effect can be explained through direct binding to an active site of biosynthetic enzymes KAT I and KAT II.

Molecular modeling data suggested that the studied loop diuretics binding site overlaps the KYN binding pocket at both KAT I and KAT II crystal structures. More specifically, the results indicated that these ligands may interact with Gly36, Phe37, Tyr101, Phe125, Gly253, and Lys255 at the KAT I site. For KAT II, we identified possible common residues involved in binding of studied diuretics and KYN (PDB ID: 2R2N), including Ile19 (A), Arg20 (A), Gly39 (A), Leu40 (A), Tyr74 (A), Leu293 (A) from one subunit and Tyr142 (B), Ser143 (B), Asn202 (B), Tyr233 (B), Phe355 (B), Phe387 (B), and Arg399 (B) from the opposite subunit. It may suggest that they may inhibit the production of KYNA through direct binding to an active site of the biosynthetic enzyme. Since tested loop diuretics inhibit KYNA synthesis only in high concentrations, it also can be postulated that especially ethacrynic acid exerts its action through a competitive inhibition, and do not impact the enzymatic Vmax.

Dualism of tryptophan metabolites, both on molecular and clinical levels, disallows their unequivocal categorization as toxic agents [39]. However, in a recently published study chronic KYN infusion in male Sprague Dawley rats resulted in mean arterial pressure elevation, decreased glomerular filtration rate (GFR), and histological patterns of kidney injury (mild proteinaceous casts and interstitial fibrosis in the medulla), suggesting that KYN and its metabolites may affect kidney function even in healthy animals [40].

It is generally accepted that, in the brain, KYNA acts mostly as a neuroprotective agent. Some data suggested that KYNA may also prevent kidney damage, e.g. in an animal model of heat stroke [41], or in a model of ischemia reperfusion-induced kidney injury [42]. However, several reports linked high KYNA levels in the serum with multiple negative effects, including endothelial damage and hypercoagulability [43], leucocyte recruitment to vascular endothelium [44], or hyperhomocysteinemia [45]. KYNA is claimed to be a protein-bound uremic toxin [46], which excretion is highly dependent on the activity of transporters localized on the basolateral membrane of the renal proximal tubule, mainly human organic anion transporter 1 (hOAT1) and human organic anion transporter 3 (hOAT3) [47] and MRP4 [48]. On the molecular level, many effects of KYNA result from the stimulation of AhR, subsequent activation of the immune system, and higher cardiovascular risk [49]. Other metabolic effects of KYNA, including inhibition of UDP-glucuronosyltransferases and mitochondrial succinate dehydrogenase activity in immortalized renal proximal tubule epithelial cells have been reported [50]. In the animal model of renal insufficiency [51] and in CKD patients [52], KYNA clearance was impaired, proportionally to the kidney function decline. Its serum level correlated with inflammatory parameters, in particular with high-sensitivity C-reactive protein and soluble tumor necrosis factor (TNF)-receptor-1 [53].

KYNA was implicated in the pathogenesis of atherosclerosis and suggested to accelerate endothelial damage in patients with kidney insufficiency [43]. Correlations between serum KYNA and cellular adhesion molecules [43, 54], or with coagulation activation markers were shown [55, 56]. In patients with atrial fibrillation [57] or subjected to hemodialysis [58], KYNA correlated with aortic stiffness, an echocardiographic marker of endothelial dysfunction. In peritoneal dialysis patients, higher KYNA levels correlated with hyperhomocysteinemia and the occurrence of cardiovascular disease [45]. However, a direct cause-and-effect relationship between KYNA and above mentioned conditions has not been proven.

Levels of protein-bound toxins, such as KYNA, are mainly dependent on residual kidney function, and cannot be efficiently decreased during standard kidney replacement therapy techniques [59, 60]. Thus, searching for other methods of lowering uremic toxins concentration, namely by inhibition of their synthesis is of special interest in delaying kidney damage progression [61]. Loop diuretics offer such an opportunity by decreasing KYNA production in the kidney.

Although data on the role of loop diuretics in kidney injury remain controversial, beneficial effects of these drugs on the preservation of kidney function were suggested. Furosemide significantly attenuated medullary thick ascending limb (mTAL) and proximal straight tubule damage in the isolated perfused rat kidney [62]. This is of special importance in the presence of limited oxygen supply, since lowered reabsorption by mTAL cells reduces their oxygen demand and improves their survival. Furosemide was also shown to decrease the ischemia reperfusion-induced apoptosis in rats, attenuate the expression of apoptosis-related genes, and upregulate Akt phosphorylation, involved in cellular survival [63]. Torasemide inhibits aldosterone binding to its cytoplasmatic receptor [64]. Moreover, in streptozotocin-induced diabetic nephropathy in rats, torasemide reduced the expression of mineral corticosteroid receptor and fibrosis-related proteins, which diminished kidney damage [65]. This effect was also observed after furosemide use [65], suggesting other mechanisms of kidney protection beyond the anti-aldosterone effect.

Lowering KYNA production may provide a beneficial effect on complications related to kidney injury. Stimulation of the KYN pathway towards KYNA and increasing its brain levels may be neuroprotective, but, paradoxically, excessive KYNA synthesis in the brain may negatively impact cognition [66]. In fact, cognitive dysfunction is common among patients with CKD and was related to lower KYNA clearance, independently from the estimated GFR (eGFR) [67].

KAT activity inhibition represents an intriguing strategy for dementia treatment [17]. In this context, loop diuretics, such as KYNA synthesis inhibitors, offer a new therapeutic option for cognitive decline treatment. Interestingly, it was already reported that furosemide inhibited cellular damage and cytokine production induced by lipopolysaccharide in microglial cells, pointing to an anti-inflammatory effect of this loop diuretic [68]. Similar suggestions are coming from human studies, in which bumetanide and furosemide use was recently associated with a significantly lower risk of Alzheimer's disease (odds ratio 0.23 and 0.42, respectively) [69].

On the other hand, considering that KYNA was shown to attenuate kidney failure in animal models [41, 42], loop diuretics administration can be considered potentially nephrotoxic. Indeed, loop diuretic-related impairment of kidney function was observed. Furosemide (5–10 mmol/l), and ethacrynic acid (0.1–5.0) mmol inhibited energy metabolism and ion transport in rat kidney cortex mitochondria [70]. Similarly, in the animal model of ischemia–reperfusion AKI, furosemide impaired oxygen consumption and structural damage of the kidney [14]. In human studies, loop diuretics increased the risk of AKI and lowered the chance of kidney function recovery [71]. However, other factors, including previous surgery or pretreatment with RAAS inhibitors may contribute to an increased risk of AKI among patients receiving furosemide [72].

The diagnostic aspect of the presented results should be also considered. The serum levels of KYNA increases faster than that of creatinine in the experimental cisplatin-induced AKI. Therefore, KYNA measurement may be superior to creatinine for the detection of kidney function decline [48].

In adults without significant kidney dysfunction, KYNA clearance had the strongest association with future eGFR decline [73]. Thus, inhibition of KYNA synthesis by loop diuretics could serve as a diagnostic test of kidney function. KYNA level analysis would be especially valuable during AKI when rapidly progressing kidney damage cannot be adequately reflected by standard laboratory parameters. Indeed, measurements of kynurenines were already used to predict responsiveness to RAAS inhibitors. In diabetic kidney disease patients, a higher KYN/tryptophan ratio correlated with the degree of albuminuria and clinical response to angiotensin II type 1 receptor blockers (ARBs) [74]. Similarly, KYN levels were significantly lower in CKD patients receiving RAAS inhibitors [75].

The limitations of the study are related to the fact that the inhibitory effect towards KYNA was displayed by the investigated compounds at high concentrations. In the clinical scenario, therapeutic levels range from 0.32 µmol/l for ethacrynic acid, through 1.49–7.75 µmol/l for torasemide and 18.14–30 µmol in the case of furosemide [76–78]. However, it cannot be excluded that loop diuretics may achieve concentrations much higher and comparable to those evaluated here locally, in the kidney. Indeed, loop diuretics in a high micromolar up to the millimolar range of concentrations were shown to affect the release of renin [79, 80] and modulate the loop of Henle’s function [81] and organic anion transporter 1 (OAT1) activity [82]. In healthy volunteers, torasemide’s concentration reached up to 555 µmol/l [83, 84].

Furthermore, while the in vitro findings are compelling, the lack of in vivo studies limits the immediate applicability of presented results to clinical settings. Future animal model research could help validate these findings in a living organism. The docking simulations suggest potential binding sites and interactions but do not confirm these interactions in a physiological context.

## Conclusions

Our study indicates a novel mechanism of action of loop diuretics, i.e. a decrease of KYNA production in rat kidney in vitro. This effect seems to be in part mediated by the inhibition of KAT I and KAT II, exerted at the active site of both enzymes. Further studies are warranted to assess the role of loop diuretics in the modulation of renal KYN pathway and its potential clinical significance.

## Data Availability

The datasets generated and analyzed during the current study are available from the corresponding author upon reasonable request.

## Abbreviations

AhR: Aryl hydrocarbon receptor
AKI: Acute kidney injury
ARB: Angiotensin II type 1 receptor blocker
CKD: Chronic kidney disease
DMSO: Dimethyl sulfoxide
eGFR: Estimated glomerular filtration rate
GFR: Glomerular filtration rate
GPR35: G protein-coupled receptor 35
HCAR: Hydroxycarboxylic acid receptor 3
hNPT4: Human sodium phosphate transporter 4
hOAT1: Human organic anion transporter 1
hOAT3: Human organic anion transporter 3
HPLC: High-performance liquid chromatography
KAT: Kynurenine aminotransferase
KYN: Kynurenine
KYNA: Kynurenine aminotransferase
MRP4: Multidrug resistance protein 4
mTAL: Medullary thick ascending limb
NKCC2: Sodium/potassium/chloride cotransporter 2
OAT1: Organic anion transporter 1
OAT4: Organic anion transporter 4
RAAS: Renin-angiotensin-aldosteron system
TNF: Tumor necrosis factor
